# UV Pretreatment Impairs the Enzymatic Degradation of Polyethylene Terephthalate

**DOI:** 10.3389/fmicb.2020.00689

**Published:** 2020-04-28

**Authors:** Patricia Falkenstein, Daniel Gräsing, Pavlo Bielytskyi, Wolfgang Zimmermann, Jörg Matysik, Ren Wei, Chen Song

**Affiliations:** ^1^Institut für Analytische Chemie, Universität Leipzig, Leipzig, Germany; ^2^Institut für Biochemie, Universität Leipzig, Leipzig, Germany

**Keywords:** solid-state NMR, chain dynamics, surface crystallinity, polyester hydrolases, plastic recycling

## Abstract

The biocatalytic degradation of polyethylene terephthalate (PET) emerged recently as a promising alternative plastic recycling method. However, limited activity of previously known enzymes against post-consumer PET materials still prevents the application on an industrial scale. In this study, the influence of ultraviolet (UV) irradiation as a potential pretreatment method for the enzymatic degradation of PET was investigated. Attenuated total reflection Fourier transform infrared (ATR-FTIR) and ^1^H solution nuclear magnetic resonance (NMR) analysis indicated a shortening of the polymer chains of UV-treated PET due to intra-chain scissions. The degradation of UV-treated PET films by a polyester hydrolase resulted in significantly lower weight losses compared to the untreated sample. We also examined site-specific and segmental chain dynamics over a time scale of sub-microseconds to seconds using centerband-only detection of exchange, rotating-frame spin-lattice relaxation (*T*_1_*_ρ_*), and dipolar chemical shift correlation experiments which revealed an overall increase in the chain rigidity of the UV-treated sample. The observed dynamic changes are most likely associated with the increased crystallinity of the surface, where a decreased accessibility for the enzyme-catalyzed hydrolysis was found. Moreover, our NMR study provided further knowledge on how polymer chain conformation and dynamics of PET can mechanistically influence the enzymatic degradation.

## Introduction

Polyethylene terephthalate (PET) is one of the most widely used plastic type, especially as packaging material for the food industry and as synthetic fibers for the textile industry (World Economic Forum, 2016; Geyer et al., 2017; PlasticsEurope, 2019). Due to its low weight, high strength, optical transparency and low CO_2_ permeability, PET is particularly suitable for the production of beverage bottles (Webb et al., 2013). In general, due to their versatile applicability and low manufacturing costs, plastics have become an indispensable part of our daily lives (Andrady and Neal, 2009), leading to a continuous increase in production which in 2018 amounted to 359 million metric tons worldwide (PlasticsEurope, 2019). However, without appropriate treatment, plastic waste can persist in nature for centuries (World Economic Forum, 2016) and accumulate as hazardous pollution causing a serious environmental crisis (Rochman et al., 2013). Hence, reduction of plastic consumption as well as recycling of in-use polymer materials have been proposed to be proper approaches to solve the plastics pollution within the framework of a sustainable circular economy (Hopewell et al., 2009).

For chemical recycling of PET, polymer chains are broken down into their monomers which can be used to produce virgin plastics or synthetic chemicals (Geyer et al., 2016). However, high temperatures and pressures as well as toxic chemicals are usually required for chemical PET recycling, making this process cost- and energy-intensive (World Economic Forum, 2016). Alternatively, biocatalytic degradation of PET which was shown to function under mild conditions in the absence of harmful chemicals, emerged as an option (Wei and Zimmermann, 2017; Kawai et al., 2019; Salvador et al., 2019; Bollinger et al., 2020). Compared to the mesophilic PET hydrolases, e.g., the PETase from *Ideonella sakaiensis* (Yoshida et al., 2016; Han et al., 2017; Austin et al., 2018), thermophilic microbial enzymes are advantageous to the hydrolysis of the ester bonds in PET due to the fact that polymer chains are more easily accessible near its glass transition temperature (*T*_g_) between 75 and 79°C (Ronkvist et al., 2009; Wei and Zimmermann, 2017; Wei et al., 2019a, b). However, long-term incubation in the *T*_g_ range is mandatory for PET degradation using the most active PET hydrolase identified so far (Wei and Zimmermann, 2017). Consequently, increased crystallinity of the polymers as a result of physical aging at this condition has been shown to hamper the enzymatic PET degradation (Wei et al., 2019a). Very recently, distinct conformations of PET segments derived by computational modeling and NMR experiments have provoked scientific discussions about the exact molecular mechanism of enzymatic PET degradation (Austin et al., 2018; Joo et al., 2018; Wei et al., 2019b). This knowledge would facilitate further exploration and engineering of enzyme variants toward enhanced PET hydrolytic activity, as well as of feasible pretreatment approaches to lower the degradation obstacle in terms of the polymer substrate.

UV light, an electromagnetic radiation in the 100-to-380-nm wavelength range, can induce photodegradation of recalcitrant polyolefins by generating free radicals (Ammala et al., 2011) and consequently facilitate the subsequent microbial degradation (Albertsson et al., 1987). PET can also absorb UV light as evidenced by photodegradation by sunlight under natural conditions leading to discoloration and brittleness on a macroscopic level (Day and Wiles, 1972b). These surface effects are likely to be associated with the cleavage of polymer chains resulting in a decrease of molecular weight and an increase of the amount of carboxylic acid end groups, accompanied by the evolution of volatile products like CO and CO_2_ (Day and Wiles, 1972a,b,c; Blais et al., 1973). Moreover, PET structural and morphological changes caused by UV irradiation have been reported previously (Fechine et al., 2002). Enzymatic PET degradation is dependent on the mobility of the amorphous regions as well as on the degree of crystallinity (Vertommen et al., 2005; Ronkvist et al., 2009; Wei et al., 2019a). Therefore, it is worthwhile to determine whether UV pretreatment has an effect on the dynamics of the shortened polymer chains and could possibly accelerate the biocatalytic degradation of PET.

In this study, we identified changes in enzymatic degradability and chain dynamics of PET caused by UV irradiation. The UV-induced PET chain scissions were quantified by both ATR-FTIR and ^1^H NMR spectroscopic techniques. The biodegradability of PET before and after UV treatment was assessed using the thermophilic polyester hydrolase LC-cutinase (LCC) originated from a plant-containing compost (Sulaiman et al., 2012). To examine the UV-induced changes in crystallinity and chain dynamics over a time scale of sub-microseconds to seconds, four different types of solid-state NMR experiments, centerband-only detection of exchange (CODEX), *T*_1_*_ρ_*, dipolar chemical shift correlation (DIPSHIFT), and cross polarization (CP) were conducted at both 30 and 70°C. The data contribute to an extended understanding of the exact enzymatic degradation mechanism at a molecular level.

## Materials and Methods

### PET Samples

**FIGURE 1:**
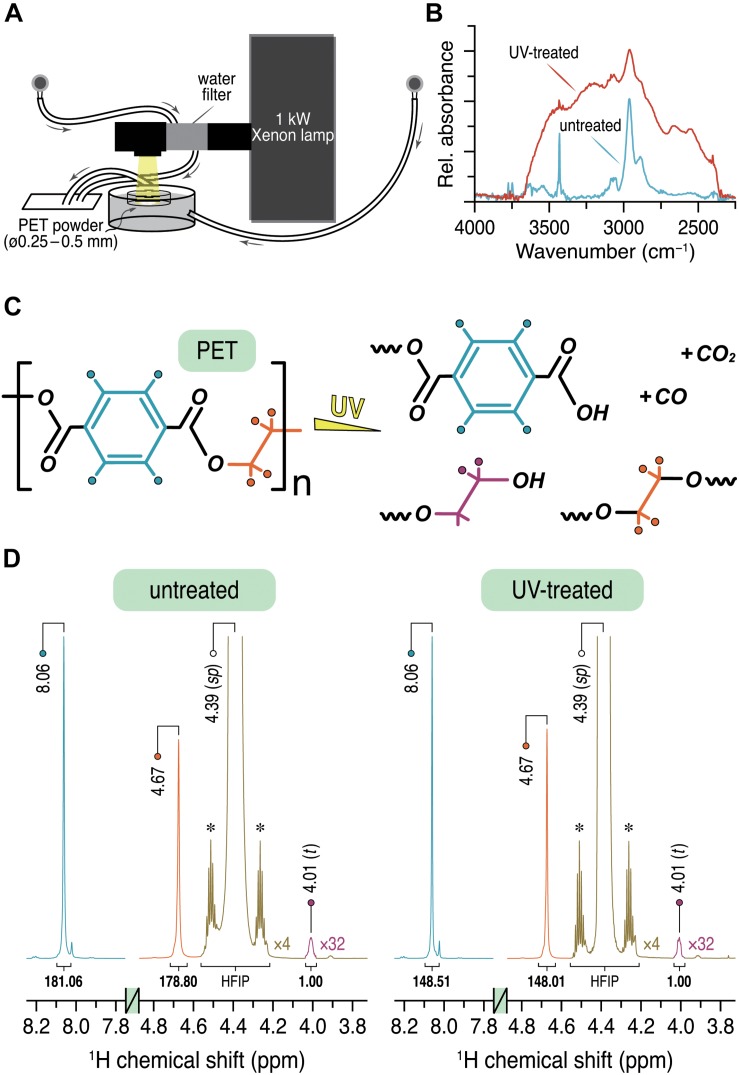
**(A)** Experimental setup for the UV irradiation of the PET powder sample. **(B)** ATR-FTIR spectra of the PET powder sample before (cyan) and after (red) UV treatment showing only the characteristic region from 2250 to 4000 cm^–1^. **(C)** Proposed products obtained by UV irradiation of PET. **(D)** ^1^H solution NMR spectra of untreated and UV-treated PET showing the relevant spectral regions from 3.8 to 4.8 ppm and 7.8 to 8.2 ppm. The relative integral ratios are given below the respective signals with the CH_2_ signal at 4.01 ppm as the unit. Intensities of the HFIP signal (green) and the CH_2_ signal (purple) are increased by a factor of 4 and 32, respectively. The ^13^C satellites of the parent HFIP signal are marked with asterisks.

For the enzymatic degradation and solution NMR experiments, amorphous PET films with a thickness of 250 μm (Goodfellow, Ltd., Bad Nauheim, Germany, product number ES301445) were used. The PET film was cryogenically ground to powder with a particle size (Ø) ranging from 0.25 to 0.5 mm for solid-state NMR measurements. UV irradiation of the PET samples was carried out over 14 days using a 1-kW xenon arc lamp (Müller GmbH Elektronik-Optik, Moosinning, Germany) which generated an emission spectrum similar to sunlight with a cut-off in the UV region at ca. 250 nm. Due to the heating effect, the IR radiation was filtered out by using a water filter. A water bath was used for further cooling the samples during irradiation (Figure 1A). The sample temperature monitored with an IR thermometer was kept below 45°C throughout the irradiation process.

### ATR-FTIR Measurements

ATR-FTIR spectra were recorded with a Bruker Vector 22 FTIR spectrometer assembled with ATR accessory using a wavenumber range of 500–4000 cm^–1^ with a resolution of 4 cm^–1^.

### ^1^H Solution NMR Measurements

^1^H NMR experiments were performed with a Bruker DRX-600 NB spectrometer equipped with a 5-mm TXI probe (Rheinstetten, Germany) at a read-out temperature of 27°C. The optimized ^1^H 90° pulse length was 9.0 μs. The data were recorded with 1 or 2 k scans and a recycle delay of 10 s. An exponential line broadening of 0.2 Hz was applied prior to data processing. The baseline was corrected manually afterward. Processed data were further analyzed using MestReNova 12.0.0 (Mestrelab Research S.L., Santiago de Compostela, Spain). Both untreated and UV-treated PET samples were dissolved in hexafluoroisopropanol (HFIP, ≥ 99%, Carl Roth GmbH + Co. KG, Karlsruhe, Germany) and stored at room temperature for 5 days before measurement. The filtered PET solutions with a final concentration of approximately 14.3 μg/μL were pipetted into a 5-mm NMR tube and mixed at a ratio of 1:16 with chloroform-*d* (CDCl_3_, 99.8% D, ARMAR AG, Döttingen, Switzerland) containing 0.03% (v/v) tetramethylsilane (TMS) as an internal standard (δ^H^, 0.00 ppm).

### Enzymatic Degradation Tests

Untreated and UV-treated PET films were cut into chips of 3 cm × 0.5 cm with an average weight of 44.7 and 44.3 mg, respectively. One PET chip was placed in a 2-mL reaction vial containing 1.8 mL of K_2_HPO_4_/Cl (1M, pH = 8.0) and 100 μg purified LC-cutinase, corresponding to a concentration of 2 μM or 1.2 nmol/cm^2^ of PET film surface area. LC-cutinase was expressed in *E. coli* BL21(DE3) and purified by immobilized metal ion chromatography similarly as previously reported (Schmidt et al., 2016). Degradation was performed by shaking the reaction vials on a thermoshaker TS1 (Biometra, Göttingen, Germany) at 70°C and 1000 rpm for 24 h. The reaction was stopped by cooling the samples in an ice bath, followed by withdrawing and washing the PET films sequentially with 0.1% SDS, ultrapure water and 70% ethanol. The PET films were dried at 50°C for > 24 h before they were subjected to gravimetric determination of the weight loss.

### Solid-State NMR Measurements

Solid-state NMR experiments were performed with a Bruker AVANCE III 400 WB spectrometer (Rheinstetten, Germany) equipped with a 4-mm double resonance magic-angle spinning (MAS) probe. For all measurements approximately 80 mg of PET powder was packed into a 4-mm zirconia rotor. Prior to NMR acquisition, *in situ* incubation of the PET sample was performed for at least 12 h to avoid spectral changes during the experiments caused by physical aging (Hutchinson, 1995). All experiments were carried out at an ambient temperature of 30°C as well as at 70°C slightly below the *T*_g_ of PET.

^1^H to ^13^C magnetization transfer was achieved by using linear 70–100% ^1^H-ramped CP (Schaefer and Stejskal, 1976; Metz et al., 1994) with a contact time of 2 ms and a ^13^C r.f. lock field of 67 kHz, fulfilling the Hartmann–Hahn condition. Heteronuclear decoupling during acquisition was achieved with swept-frequency two-pulse phase modulation (Thakur et al., 2006; Vinod Chandran et al., 2008) at ^1^H r.f. field of 100 kHz. Optimized ^1^H and ^13^C 90° pulse lengths were 2.5 and 3.0 μs, respectively. ^13^C CP/MAS spectra were collected at a spinning frequency of 12.5 kHz with 2 k scans and a recycle delay of 2.5 s. Overall, 1604 data points were recorded. For the data processing, an exponential window function and zero filling to 8192 data points was applied prior to Fourier transformation.

DIPSHIFT (Hong et al., 1997) data were collected with a recycle delay of 4 s. The ^1^H–^1^H homonuclear dipolar decoupling during the *t*_1_ evolution period was accomplished by using the phase modulated Lee–Goldburg (PMLG5) (Vinogradov et al., 2001) scheme. Each PMLG5 block consisted of 10 pulses with the following phases: 339.22, 297.65, 256.08, 214.51, 172.94, 352.94, 34.51, 76.08, 117.65, and 159.22° (m5m shape in Top-Spin 3.2 library). The optimized PMLG5 pulse was 2.08 μs with 80 kHz r.f. amplitude. For different experiments the number of scans per increment was varied between 768 and 1168. An exponential window function was applied and the data was zero-filled to 8192 points prior to Fourier transformation. The rigid-limit values for CH and CH_2_ groups were determined experimentally by measuring DIPSHIFT curves for crystalline *bis*(2-hydroxyethyl) terephthalate (BHET) and fitting them in a site-specific manner. All DIPSHIFT curves were fitted with SIMPSON simulations (Bak et al., 2000) taking into account the PMLG scaling factor of 0.5 which was determined by observing the *J*-splitting of the CH and CH_2_ groups of adamantane under PMLG5-decoupling using the same conditions.

CODEX (deAzevedo et al., 1999) data were recorded with a recycle delay of 2.5 s. At 30°C, 2k scans were accumulated, while at 70°C the number of scans was around 4 k (in multiples of 128). An exponential window function was applied and the data points were zero-filled to 8192 prior to Fourier transformation. A *t*_z_ filter time of 1.28 ms and mixing times (*t*_m_) between 0.02 and 1.2 s were employed. The sum of the preparation and refocusing time was 10 *t*_r_ (*N* = 10). An extended phase cycle (Reichert et al., 2001) was used for proper cancelation of unwanted transverse components during *t*_m_ and *t*_z_ under fast MAS.

*T*_1_(^1^H) relaxation times were measured with 392 and 784 scans for the untreated and UV-treated samples, respectively. A 66 kHz ^1^H spin-lock field and a recycle delay of 4 s were used. The length of the spin-lock pulse was incremented in 13 steps varying between 0.01 and 50 ms. An exponential window function was applied in the ^13^C dimension for processing while the data points were zero-filled up to 16 and 2048 data points in the ^1^H and ^13^C dimension, respectively. *T*_1_(^1^H) relaxation times were obtained by least-squares fitting of the normalized peak intensities to a single exponential:

(1) $$\frac{M\,\left(\tau \right)}{M\,\left(0\right)}=e^{-\frac{\tau }{T_{1\,\rho }}}$$

Here, *M*(0) and *M*(*τ*) represent the initial intensity and the intensity at the applied spin-lock time *τ*, respectively.

For *T*_1_(^1^H) and CODEX analysis OriginPro 8G (OriginLab Corporation, Northampton, MA) was used. Spectral fitting was done with MestReNova 12.0.0 (Mestrelab Research S.L., Santiago de Compostela, Spain). ^13^C chemical shifts were externally referenced to the COO^–^ signal of solid tyrosine hydrochloride at 172.1 ppm.

## Results

### ATR-FTIR and ^1^H Solution NMR Analysis of PET Chain Scission Caused by UV Irradiation

To determine the chain scissions of PET by UV light (Figure 1A), surface sensitive ATR-FTIR measurements (Day and Wiles, 1972b, c; Blais et al., 1973; Fechine et al., 2004) were performed. The untreated PET sample (Figure 1B, cyan) showed the characteristic absorption bands, as expected. The band at 3060 cm^–1^ can be assigned to the aromatic C–H stretching and the corresponding aliphatic C–H stretching modes are represented by the bands at around 2960 and 2880 cm^–1^ (see also Holland and Hay, 2002). The first overtone of the fundamental C=O absorption at 1710 cm^–1^ (Supplementary Figure 1) appeared at ∼3430 cm^–1^ (Day and Wiles, 1972b). The same bands can be identified in the spectrum of the PET sample exposed to UV light for 14 days (Figure 1B, red). However, clear differences in the range between 2250 and 3700 cm^–1^ were observed. The broad absorption bands centered at around 3500 and 3250 cm^–1^ can be attributed to O–H stretching vibrations from the –COOH and –OH functional groups, respectively (Day and Wiles, 1972b). The shoulders at 2660 and 2550 cm^–1^ most likely originate from overtones and combination bands, characteristic for dimeric carboxylic acids (Schrader and Meier, 1974; Günzler and Gremlich, 2003). These distinctive absorption bands can therefore be ascribed to an increased carboxyl end group content in the UV-treated PET sample relative to the untreated one. It is also clear that the formation of additional carboxyl end groups was caused by the breakage of the ester C–O bonds in the main chain (Figure 1C), while the concentration of carboxyl end groups is too low and therefore not detectable in the corresponding ATR-FTIR spectrum of the untreated sample.

To quantify the intra-chain scissions of the UV-treated PET, the degree of polymerization (DP) was determined by ^1^H NMR spectroscopy similarly as described before (Wei et al., 2019a). Both ^1^H NMR spectra display two sharp singlets with almost identical integrals (< ± 1%) at 8.06 and 4.67 ppm (Figure 1D) which can be readily assigned to the four protons of the terephthalate ring and the oxyethylene units in the main chain, respectively (Kenwright et al., 1999; de Ilarduya and Muñoz-Guerra, 2014). A much weaker signal with δ^H^ of 4.01 ppm (2H, *t*, ^3^*J ≈* 4.5 Hz) corresponds to the methylene protons in *α*-position to the hydroxyl end group of the PET chain (Figure 1C). The DP value was determined according to the relative integral ratios of these three signals. Assuming that each PET chain possesses a single hydroxyl end group, a DP of 181.7 ± 1.6 was obtained for the untreated sample, while the UV-treated sample showed a DP value of 149.7 ± 0.4 with a reduction of 17.6%. Considering the molecular weight of 192.17 g/mol of a single PET repeating unit, the two DP values for the untreated and the UV-treated samples corresponded to an average molecular weight of about 35000 and 29000 g/mol, respectively.

### Enzymatic Degradation of UV-Treated PET Films

**FIGURE 2:**
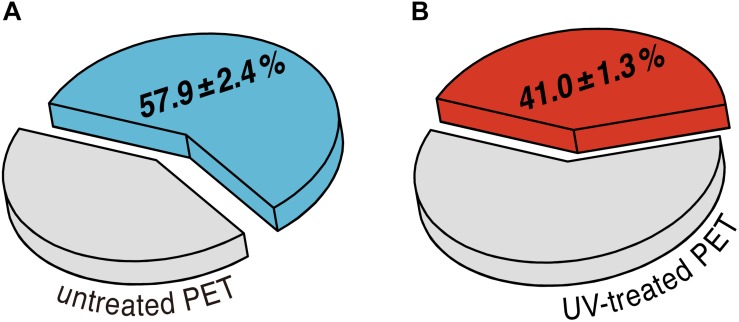
Average percentage of weight loss of **(A)** untreated and **(B)** UV-treated PET films after enzymatic degradation performed at 70°C for 24 h. The standard deviations are obtained from at least triplicate experiments.

To assess the effect of UV pretreatment on the enzymatic degradation of PET, we performed hydrolytic degradation of the untreated and the UV-treated PET films at 70°C for 24 h using purified recombinant LC-cutinase. The weight loss of the PET films after enzymatic hydrolysis was determined gravimetrically to evaluate the degradation performance. According to a previous study, weight loss of amorphous PET films could be quantitatively correlated with the release of degradation monomers including terephthalic acid, ethylene glycol (EG), mono(2-hydroxyethyl) terephthalate (MHET) and BHET (Barth et al., 2015). As shown in Figure 2, an absolute weight loss of 26.1 ± 0.5 mg (57.9 ± 2.4%) and 18.2 ± 0.9 mg (41.0 ± 1.3%) was determined for the control sample and the UV-treated PET sample, respectively, as a result of 24 h enzymatic hydrolysis.

### ^13^C CP/MAS Experiments to Determine the Degree of Crystallinity in PET Before and After UV Irradiation

**FIGURE 3:**
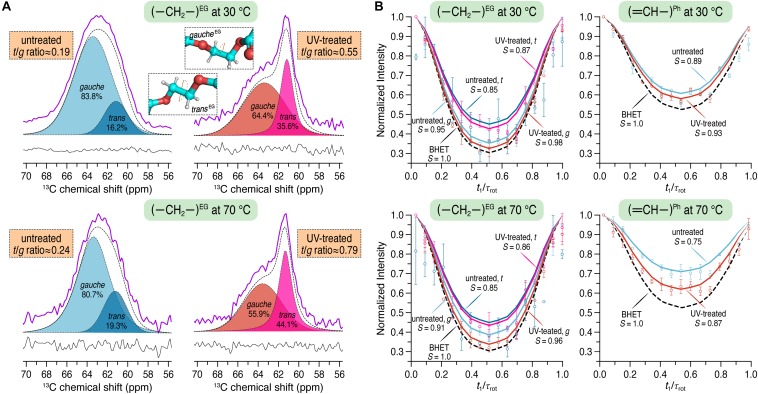
**(A)** ^13^C CP/MAS spectra showing only the characteristic spectral region for the carbons of the ethylene glycol (EG) unit before (*left*) and after (*right*) UV irradiation at 30 and 70°C using a spinning frequency of 12.5 kHz. The *trans* and *gauche* contents were quantified by fitting a Voigt function to the experimental spectra (purple). *Trans* and *gauche* conformations were assigned according to Gabriëlse et al. (1994). **(B)**^ 1^H–^13^C dephasing curves for the EG (*left*) and the phenylene unit of PET (*right*) obtained from DIPSHIFT experiments at 30 and 70°C using a spinning frequency of 8 kHz. DIPSHIFT curves were fitted by using 17 and 16 points, respectively, for the ethylene and the aromatic C-H groups.

^13^C CP/MAS spectra were recorded to determine the *trans*/*gauche* (*t*/*g*) ratio of the PET samples before and after UV irradiation. Amorphous PET exists as a mixture of *t* and *g* conformers, whereby the *g* content usually significantly exceeds the *t* content. On the contrary, crystalline PET consists of 100% *t* conformation. The average OC–CO torsion angle of *t* and *g* states is about 180 and ± 60°, respectively (Schmidt-Rohr et al., 1998). A quantification for the *t/g* ratio was done by deconvolution of the ethylene carbon signals (Figure 3A), composed of both *t* and *g* conformations, corresponding to the crystalline and the amorphous phase of PET, respectively (Gabriëlse et al., 1994; Choudhury et al., 2012). For the untreated PET sample, a *t/g* ratio of 0.19 ± 0.03 was calculated from the spectra at 30°C and a value of 0.23 ± 0.06 was determined at 70°C. After UV treatment the *t/g* ratio of the PET sample was increased at both 30 and 70°C with the values of 0.49 ± 0.08 and 0.67 ± 0.16, respectively. The conformational changes of the PET chains associated with the UV treatment were also evident from the splitting of the carbonyl signals after UV irradiation (data not shown).

### Site-Specific and Segmental PET Chain Dynamics

To analyze dynamical changes of untreated and UV-treated PET over a time scale of sub-microseconds to seconds, three different solid-state NMR experiments were conducted.

The DIPSHIFT experiment is used to quantify localized dynamics of proteins or polymers on the sub-microseconds time scale (Huster et al., 2001; deAzevedo et al., 2008; Ivanir-Dabora et al., 2015). Here, the order parameters *S* (ranging from 0 to 1 representing isotropic motion and a rigid limit, respectively) were determined at both 30 and 70°C for the PET samples before and after UV irradiation. The ^1^H–^13^C dephasing curves extracted from the DIPSHIFT experiments including the calculated order parameters are shown in Figure 3B. The rigid limit with the order parameter *S* = 1 is represented here by BHET.

For the untreated PET sample an order parameter of *S* = 0.89 and 0.75 was obtained for the aromatic C–H group at 30 and 70°C, respectively. In the UV-treated PET sample, however, the phenylene unit revealed larger order parameters at both temperatures (*S* = 0.93 and 0.87 at 30 and 70°C, respectively). It is clear that more pronounced chain motions are detected for both samples at the higher temperature. Furthermore, after UV irradiation, the phenylene unit shows reduced mobility on the sub-microseconds time scale at both temperatures with larger order parameters (*S* = 0.93 vs. 0.89 at 30°C, and *S* = 0.87 vs. 0.75 at 70°C). For the ethylene unit, the order parameters *S* for both *t* and *g* conformations were calculated. After UV treatment an order parameter of *S* = 0.98 was found for the *g* conformation, slightly larger than that of the untreated sample at 30°C (*S* = 0.95). At 70°C, *S* = 0.91 and 0.96 were observed before and after UV irradiation, respectively. For the *t* conformation of the ethylene unit *S* = 0.85 and 0.87 were determined for the untreated and UV-treated PET at 30°C, respectively. The order parameter obtained at 70°C for the untreated PET sample was *S* = 0.85 and 0.86 for UV-illuminated PET. The overall increased order parameters clearly indicate that the ethylene unit of PET is getting less mobile after UV degradation.

In addition, we also employed the CODEX experiment for characterization of slower segmental reorientations of the phenylene unit over a time scale of milliseconds to seconds (deAzevedo et al., 1999) which was reported to undergo ring-flip motions in PET about its 1,4-axis (Wilhelm and Spiess, 1996; Choudhury et al., 2012). By plotting the normalized exchange intensity which is the ratio of pure-exchange CODEX intensity to the reference intensity, against the different mixing times (*t*_m_), the correlation time of the reorientation can be obtained (deAzevedo et al., 1999). In the case of PET, the curve was characterized by the following equation representing a stretched exponential (Choudhury et al., 2012):

(2) $$\Delta \,S/S_{0}=E_{\infty }\,\left(1-e^{-{\left(t_{\text{m}}/\tau_{\text{c}}\right)}^{\beta }}\right)$$

Here, *E* is the final exchange intensity and β is the stretch exponent. Moreover, *τ*_c_ represents the center of the correlation time distribution of the motion. Furthermore, the number *M* of equivalent orientational sites accessed by a specific carbon in the motional process as well as the fraction *f*_m_ of mobile segments can be calculated by means of *E* (deAzevedo et al., 1999):

(3) $$E_{\infty }=\frac{f_{\text{m}}\,\left(M-1\right)}{M}$$

**FIGURE 4:**
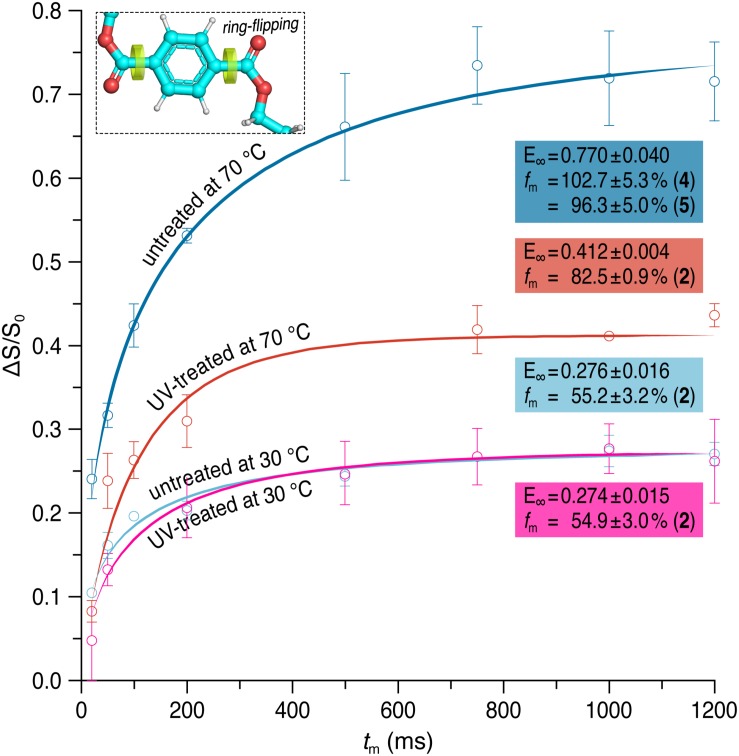
Pure-exchange CODEX signals as a function of mixing time (*t*_m_) for the protonated aromatic carbons at 30 and 70°C before (dark/light blue) and after (red/pink) UV irradiation. CODEX experiments were performed at a spinning frequency of 12.5 kHz. Solid lines represent fits of the experimental data to a stretched exponential. The final exchange intensity *E* and the fraction *f*_m_ of mobile segments is given for each curve. The number *M* of equivalent orientational sites accessed by a specific carbon in the motional process which was used for calculating *f*_m_ is shown in brackets.

The normalized pure-exchange CODEX intensities of the protonated aromatic carbons of the untreated and the UV-treated samples are plotted in Figure 4. At 70°C, a final exchange intensity of *E* = 0.770 ± 0.040 and 0.412 ± 0.004 was determined for the untreated and the UV-treated sample, respectively. At 30°C, *E* = 0.276 ± 0.016 was obtained for untreated PET and *E* = 0.274 ± 0.015 for the UV-treated PET sample. The fraction of mobile segments is *f*_m_ = 54.9 ± 3.0 and 55.2 ± 3.2% for the UV-treated and the untreated PET sample at 30°C, respectively. The results are therefore similar at a temperature of 30°C. However, at 70°C the mobile fractions are increased, with *f*_m_ = 82.5 ± 0.9% for the UV-treated sample and *f*_m_ = 96.3 ± 5.0 or 102.7 ± 5.3%, depending on the chosen value for *M*, for the untreated PET sample. The number of equivalent orientational sites (*M*) accessed by one protonated aromatic carbon is *M* = 2 for the UV-treated sample at both temperatures and for the untreated PET sample at 30°C, indicating the 180° phenylene ring flip. For the untreated sample at 70°C the value of *M* is assumed to be 4 or 5, suggesting more complex motions of the phenylene ring. Since the correlation time values are quite sensitive to curve shapes and single data points, their errors are large and these values are therefore not taken into account for evaluating dynamical changes of the PET samples (Choudhury et al., 2012). Results of the fitting of experimental data to a stretched exponential (Eq. 2) and data analysis using Eq. 3 are shown in Supplementary Table 1.

**FIGURE 5:**
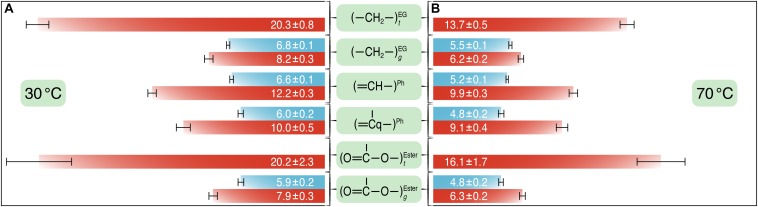
The *T*_1_*_ρ_*(^1^H) relaxation times (in milliseconds) for the different carbons of PET before (blue) and after (red) UV irradiation at 30°C **(A)** and at 70°C **(B)**. *T*_1_*_ρ_*(^1^H) experiments were performed at a spinning speed of 8 kHz with varying spin-lock pulse lengths between 0.01 and 50 ms. *T*_1_*_ρ_*(^1^H) values and corresponding error bars were obtained from the fits of the normalized peak intensities against the spin-lock time to a single exponential curve.

Furthermore, *T*_1_*_ρ_*(^1^H) relaxation times were measured for both PET samples which provide segmental information on dynamics over a time scale of microseconds to milliseconds (Schmidt-Rohr and Spiess, 1994). Since *T*_1_*_ρ_*(^1^H) experiments are susceptible to ^1^H spin diffusion which results in the averaging of relaxation times, more global information about the PET chain motions can thus be obtained (VanderHart and Garroway, 1979; Gabriëlse et al., 1994). To determine *T*_1_*_ρ_*(^1^H) times the normalized peak intensities were plotted against the spin-lock time *τ* and fitted to a single exponential for untreated and UV-treated PET. The values are summarized in Figure 5.

At 70°C, *T*_1_*_ρ_*(^1^H) relaxation times determined for both untreated and UV-treated samples were shorter than those at 30°C, indicating shorter average motional correlation times and thus faster motions, for example *T*_1_*_ρ_*(^1^H)_70__^__C_ = 5.2 ± 0.1 ms vs. *T*_1_*_ρ_*(^1^H)_30__^__C_ = 6.6 ± 0.1 ms for the protonated aromatic carbons in the untreated PET sample. After UV irradiation, the *T*_1_*_ρ_*(^1^H) values were larger at both temperatures, for example the *T*_1_*_ρ_*(^1^H) value was 12.2 ± 0.3 and 6.6 ± 0.1 ms for the protonated aromatic carbons in the UV-treated and the untreated sample at 30°C, respectively. The ethylene and carbonyl peaks were deconvoluted into amorphous and crystalline components for the UV-treated sample. For carbons in *t* conformation, the *T*_1_*_ρ_*(^1^H) times were significantly larger than those in *g* conformation (e.g., *T*_1_*_ρ_*(^1^H)*_*t*_* = 20.3 ± 0.8 and *T*_1_*_ρ_*(^1^H)*_*g*_* = 8.2 ± 0.3 for the ethylene carbons in the UV-treated sample at 30°C), indicating that crystalline regions of PET with relatively high *t* content are more rigid than amorphous regions with chains in *g* conformation. The values for the ethylene carbons in *t* and *g* conformations at 30°C are very similar to those determined by Choudhury et al. (2012) for semicrystalline PET.

## Discussion

The enzymatic degradation of post-consumer PET has emerged as an eco-friendly method for future applications in plastic recycling processes (Wei and Zimmermann, 2017). UV irradiation is a major abiotic factor responsible for the environmental plastic degradation (Gewert et al., 2015), and is thus considered as a potential pretreatment to mitigate the hurdles for biodegradation caused by polymer microstructures. Photodegradation-based pretreatment has proven to be beneficial for subsequent biodegradation of selected polyolefins (Koutny et al., 2006a, b; Restrepo-Flórez et al., 2014), however, so far rarely investigated for PET. Chain scission has been verified with PET samples exposed to sunlight for up to 42 days already in the 1970s (Day and Wiles, 1972b) providing a fundamental background for further studies. The PET samples in this study were exposed to a UV light source which shows an emission spectrum similar to sunlight, thus making it possible to mimic natural illumination conditions. We used ATR-FTIR and ^1^H solution NMR spectroscopy to analyze the PET samples after UV exposure. Both spectroscopic methods revealed an increased amount of end groups and a decreased average molecular mass from 35000 to 29000 g/mol (Figure 1D) thereby suggesting the PET sample has been partially photodegraded by the UV light.

However, the UV-treated PET samples showed an increased resistance against enzymatic degradation yielding lower weight losses of the material compared to an untreated control (Figure 2). To clarify this seeming contradiction, solid-state NMR spectroscopy is the method of choice as it provides detailed information at the atomic level. Therefore, we performed a series of solid-state NMR experiments with both the UV-treated and control PET samples. The ^13^C CP/MAS experiment was used to determine the *t*/*g* ratio of the PET samples (Figure 3A), where a higher *t* content is detected for the UV-treated PET, indicative of an increased crystallinity as a result of UV exposure. These results are consistent with a previous study which reported a 100% increase of crystallinity of a semicrystalline PET sample after exposure to sunlight for 670 days determined by density measurement and differential scanning calorimetry (Aljoumaa and Abboudi, 2016). UV degradation is known to be a surface sensitive process (Day and Wiles, 1972b; Blais et al., 1973), and accordingly, the resulting increased crystallinity is considered to affect only the surface of the sample. DIPSHIFT, *T*_1_*_ρ_*(^1^H) and CODEX experiments provide information about the dynamics of polymer chains in the untreated and the UV-irradiated PET on different time scales. Higher order parameters *S* (Figure 3B) were observed for the EG and the phenylene C–H groups after UV photodegradation, indicating that both regions became less mobile. Moreover, CODEX experiments revealed that the number of equivalent orientational sites *M* accessed by one carbon is highest for the untreated sample measured at 70°C (Figure 4), suggesting more complicated chain motions in untreated PET at this temperature. For the UV-treated sample only two-site jumps were observed at both temperatures which can be attributed to the already known 180° phenylene ring flip about the 1,4-axis indicating a partially restricted rotation at 70°C in comparison with that of the untreated sample. The *t* and *g* conformations were indistinguishable in the CODEX spectra, the lower mobility can thus be ascribed to the higher crystalline content in PET after UV irradiation, representing a more ordered system with for example a more hindered ring-flipping motion of the phenylene unit. In agreement with CODEX and DIPSHIFT data, the *T*_1_*_ρ_*(^1^H) relaxation times became larger after UV irradiation (Figure 5) which clearly indicates a reduction in segmental dynamics over a time scale of microseconds to milliseconds. The overall slower chain motions of the UV-treated PET sample can be ascribed to the change of microstructure in the surface layer. UV light can result in spontaneous chain scission in PET by oxidation and consequently shorten the polymer chains (Day and Wiles, 1972c). The shorter PET polymer chains are more prone to have fold among themselves and thus to be rearranged into inter-crystalline domains (Launay et al., 1994; Badia et al., 2012). Due to the increase in local temperature of the surface layer exposed to UV light, physical aging of short PET chains with a trend to more ordered structure thus occurred more easily. Increased crystallinity in the surface layer and consequently decreased polymer chain dynamics hampers the efficiency of biocatalytic degradation which is well-known as a surface erosion process (Ronkvist et al., 2009).

Recent studies on the mechanism of biocatalytic degradation by computational simulation and solid-state NMR indicated that the flexibility of both the polymer chain and the substrate binding cleft on the surface of the enzyme is crucial to ensure a high degradation performance against PET (Austin et al., 2018; Wei et al., 2019b). Here, for the PET sample treated with UV irradiation, an overall increased chain rigidity including the partially prohibited ring-flip motions of the phenylene units was observed. This would hamper the hydrophobic interactions between the aromatic phenylene units and the neighboring amino acids in the active site required for the binding of PET substrate (Austin et al., 2018; Wei et al., 2019b) and consequently diminish the efficiency of the enzymatic hydrolysis.

Besides the –COOH and –OH groups, the broadened C=O band at 1800–1750 cm^–1^ in the ATR-FTIR spectrum of UV-treated PET (Supplementary Figure 1) gives an indication for vinyl ester end groups (Hesse et al., 2005). Additionally, the formation of aldehyde end groups is also conceivable (see also Gewert et al., 2015). The increased complexity of the PET surface upon UV irradiation supports the original proposal of a radical-based mechanism in photo-oxidative degradation (Day and Wiles, 1972a; Gotoh et al., 2011; Gewert et al., 2015). Since the biocatalytic degradation of low-crystalline PET has been shown to follow a processive exo-mechanism (Wei et al., 2019a), the formation of these alternative end groups as a result of UV treatment would impair the recognition of the polymer chain ends by the polyester hydrolase and consequently reduce the efficiency of a biocatalytic PET degradation.

In addition to the use of purified enzymes, the biodegradation of PET has also been shown with natural and engineered microbes in pure cultures at ambient temperatures (Yoshida et al., 2016; Moog et al., 2019). Using these whole-cell based systems, the degradation performance of PET was shown to rely on the activity and amount of the PET hydrolase expressed and secreted. A colonization of the PET polymer by the microbes has been considered as a requirement for an efficient degradation by their secreted enzymes. Nevertheless, only colonization of *Ideonella sakaiensis* on amorphous PET materials has been evidenced so far in correlation with a notable polymer degradation (Yoshida et al., 2016). More recently, another study speculated that microbial biofilm formation might be more pronounced on PET materials exposed to UV radiation for 30 min (Vague et al., 2019). However, the biodegradation of PET could not be unambiguously verified by either demonstrating the release of degradation products or by a significant weight loss. Therefore, the question whether UV treatment will influence a microbial colonization of PET at ambient temperatures remains still unsolved. Our recent study has indicated that thermophilic conditions at ≥ 70°C are preferred for an efficient PET biodegradation (Wei et al., 2019b). A whole-cell based system with simultaneous microbial growth, enzyme expression and biocatalytic PET degradation at elevated temperature is a target of the ongoing research.

The UV fraction of natural sunlight has been shown to have similar aging effects on PET materials as the artificial light source used in this study (Aljoumaa and Abboudi, 2016). Concerning marine litter, the PET debris is largely coming from PET beverage bottles with a higher crystallinity than the amorphous PET used in this study. A further exposure to sunlight can be expected to render them even less biodegradable serving as a vehicle for microbial attachment rather than a substrate for the accumulation of specific consortia able to degrade and assimilate the plastic (Oberbeckmann et al., 2014, 2016).

In summary, we investigated the chain conformational and dynamic changes as well as biodegradability of amorphous PET after UV pretreatment. Analysis by various spectroscopic techniques and enzymatic degradation studies revealed the formation of a surface layer with increased crystallinity as a result of UV treatment which consequently decreased polymer chain dynamics causing significantly retarded enzymatic hydrolysis. Our findings suggest that the exposure to UV light is not a feasible pretreatment approach to enhance the following biocatalytic degradation of PET, despite the occurrence of shorter polymer chains as a result of photodegradation. Further NMR studies are needed for in-depth understanding of how the polymer chain conformation and dynamics can influence the enzymatic biodegradation of PET at the molecular level.

## Data Availability Statement

All datasets generated for this study are included in the article/Supplementary Material.

## Author Contributions

JM and CS conceived the experiments. PF, DG, PB, JM, RW, and CS designed the experiments. PF performed NMR and ATR-FTIR experiments. RW performed enzymatic degradation of PET films. PF, RW, and CS analyzed the data. WZ and JM supervised the project. PF wrote the manuscript with extensive input from WZ, JM, RW, and CS. All the authors read and corrected the manuscript.

## Conflict of Interest

The authors declare that the research was conducted in the absence of any commercial or financial relationships that could be construed as a potential conflict of interest.
