# Supplementary material for: UV Pretreatment Impairs the Enzymatic Degradation of Polyethylene Terephthalate
Source: Front Microbiol. 2020 Apr 28;11:689. doi: 10.3389/fmicb.2020.00689 (PMC7199389; doi:10.3389/fmicb.2020.00689)
Supplement: Supplementary file 1 [file Data_Sheet_1.PDF]

Supplementary Table 1. Stretched exponential fit parameters of the CODEX signal of protonated aromatic carbons in PET and mobile fractions f_m with number M of equivalent orientational sites in brackets.

Sample	E_∞	τ_c / ms	β	f_m
30 °C before UV	0.276 ± 0.016	81 ± 23	0.51 ± 0.08	0.552 ± 0.032 (M = 2)
70 °C before UV	0.770 ± 0.040	151 ± 27	0.54 ± 0.04	1.027 ± 0.053 (M = 4)<br>0.963 ± 0.050 (M = 5)
30 °C after UV	0.274 ± 0.015	107 ± 28	0.63 ± 0.13	0.549 ± 0.030 (M = 2)
70 °C after UV	0.412 ± 0.004	104 ± 23	0.82 ± 0.17	0.825 ± 0.009 (M = 2)

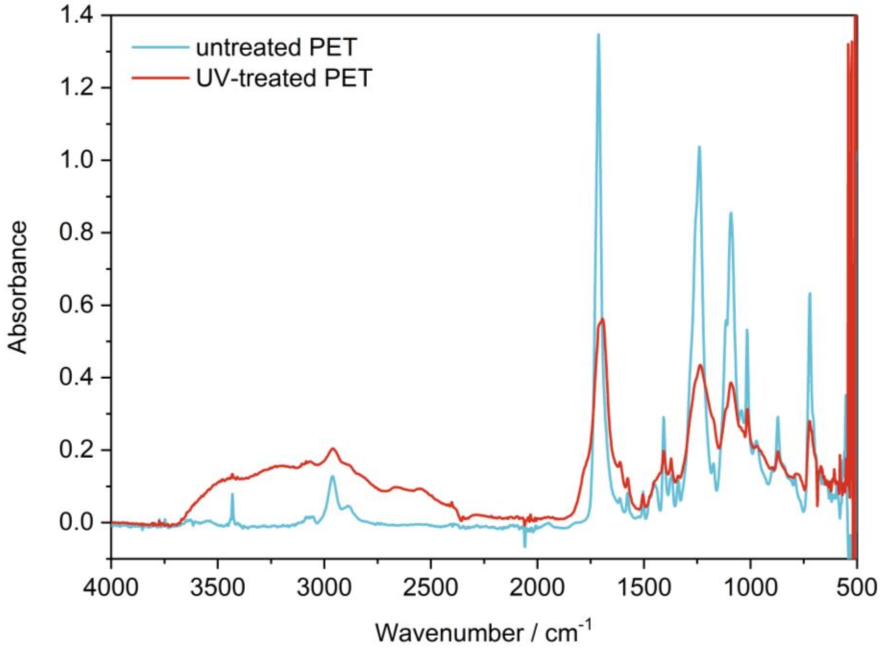

Supplementary Figure 1. ATR-FTIR spectra of the PET powder sample before (blue) and after (red) UV pretreatment.
